# Icariside II, a Broad-Spectrum Anti-cancer Agent, Reverses Beta-Amyloid-Induced Cognitive Impairment through Reducing Inflammation and Apoptosis in Rats

**DOI:** 10.3389/fphar.2017.00039

**Published:** 2017-02-02

**Authors:** Yuanyuan Deng, Long Long, Keke Wang, Jiayin Zhou, Lingrong Zeng, Lianzi He, Qihai Gong

**Affiliations:** ^1^Department of Pharmacology, Key Laboratory of Basic Pharmacology of Ministry of Education, Zunyi Medical UniversityGuizhou, China; ^2^Department of Pharmacy, Zunyi Medical UniversityGuizhou, China; ^3^Zunyi Medical and Pharmaceutical CollegeGuizhou, China

**Keywords:** Alzheimer’s disease, ICS II, beta-amyloid, neuroinflammation, apoptosis

## Abstract

Beta-amyloid (Aβ) deposition, associated neuronal apoptosis and neuroinflammation are considered as the important factors which lead to cognitive deficits in Alzheimer’s disease (AD). Icariside II (ICS II), an active flavonoid compound derived from *Epimedium brevicornum* Maxim, has been extensively used to treat erectile dysfunction, osteoporosis and dementia in traditional Chinese medicine. Recently, ICS II attracts great interest due to its broad-spectrum anti-cancer property. ICS II shows an anti-inflammatory potential both in cancer treatment and cerebral ischemia-reperfusion. It is not yet clear whether the anti-inflammatory effect of ICS II could delay progression of AD. Therefore, the current study aimed to investigate the effects of ICS II on the behavioral deficits, Aβ levels, neuroinflammatory responses and apoptosis in Aβ_25-35_-treated rats. We found that bilateral hippocampal injection of Aβ_25-35_ induced cognitive impairment, neuronal damage, along with increase of Aβ, inflammation and apoptosis in hippocampus of rats. However, treatment with ICS II 20 mg/kg could improve the cognitive deficits, ameliorate neuronal death, and reduce the levels of Aβ in the hippocampus. Furthermore, ICS II could suppress microglial and astrocytic activation, inhibit expression of IL-1β, TNF-α, COX-2, and iNOS mRNA and protein, and attenuate the Aβ induced Bax/Bcl-2 ratio elevation and caspase-3 activation. In conclusion, these results showed that ICS II could reverse Aβ-induced cognitive deficits, possibly via the inhibition of neuroinflammation and apoptosis, which suggested a potential protective effect of ICS II on AD.

## Introduction

Alzheimer’s disease, with impairment in memory and cognition as the main clinical manifestation, has become a considerable social and medical burden for causing chronic disability and high mortality among elders (Joan et al., 2013; Alzheimer’s Association, 2015). Growing evidences show that the overproduction and accumulation of neurotoxic β-amyloid peptide (Aβ) in the brain is the primary cause driving AD pathogenesis (Barnabas James, 1997; Suh and Checler, 2002; Gouras et al., 2014). While the underlying mechanisms of Aβ-induced neuronal death in AD remain unclear, accumulating studies suggest that neuroinflammation and apoptosis may be a consequence of the neurotoxic Aβ accumulation and may play a role in AD progression and pathology (Bamberger and Landreth, 2002; Paula et al., 2010). Moreover, novel agents targeting apoptosis suppression and/or inflammatory inhibition can slow the progression of cognitive dysfunction and/or accumulation of Aβ in animal models (Townsend and Domenico, 2005; Wang et al., 2012). However, the existing anti-inflammatory agents such as non-steroidal anti-inflammatory drugs (NSAIDs) have generated mixed results (Breitner et al., 2009; Potter, 2010). Thus, it is an imperative to develop new and alternative anti-inflammatory and/or anti-apoptosis therapies to be used for AD treatment.

*Herba Epimedii* (*Epimedium brevicornum* Maxim, Yinyanghuo in China), a traditional Chinese herbal medicine, has been widely used in East Asia for the treatment of cardiovascular disease, osteoporosis, erectile dysfunction and dementia (Xiao et al., 1993; Meng et al., 2005; Sze et al., 2010). ICS II, a flavonoid isolated from *Epimedii* herb, has been shown to possess a wide range of pharmacological effects on anti-tumor, anti-oxidation and anti-osteoporosis. Recently, ICS II has gain increasing interests in its anti-cancer properties, and *in vitro* and *in vivo* studies suggest ICS II displayed activity against lung carcinoma, prostate cancer, melanoma and breast cancer (Lee et al., 2009; Song et al., 2012; Wu et al., 2015). Common signaling pathways and therapeutic targets relating cancer and neurodegenerative diseases have been increasingly reported (Plunfavreau et al., 2010; Driver, 2012). A recent study showed that the anti-cancer mechanism of ICS II is its suppressive effects of pro-inflammatory cytokines in inflammatory microenvironment (Jie et al., 2016). Inflammation is a hallmark of cancer and various central nervous system disorders. Our research group and other teams have recently shown that ICS II has neuroprotective effects during cerebral ischemia-reperfusion via inflammatory inhibition and apoptosis suppression (Yan et al., 2014; Deng et al., 2016). It is implied that ICS II as an anti-inflammation agent could be potentially used to ameliorate neurodegenerative diseases. However, whether ICS II exerts protective effects on AD is still unknown.

The present study was designed to investigate whether ICS II treatment ameliorates cognitive deficits induced by hippocampal Aβ_25-35_ injection in rats. Furthermore, inflammatory responses and neuronal apoptosis were explored to elucidate the possible mechanisms underlying the protective effect of ICS II on AD.

## Materials and Methods

### Drugs and Chemicals

Icariside II (purity ≥ 98% by HPLC), purchased from Nanjing Zelang Medical Technology Corporation Ltd. (Nanjing, China), and dissolved in NS. Aβ_25-35_ was purchased from Sigma-Aldrich (St. Louis, MO, USA), dissolved in sterilized NS at the concentration of 2 μg/μL, and then incubated at 37°C for 7 days before injection to make the state of aggregation (Pike et al., 1995; Nie et al., 2010).

### Animals

Sixty adult male Sprague-Dawley rats (3 months old, 250 to 280 g) were provided by the Experimental Animal Center of Third Military Medical University (SPF-grade, Certificate NO. SCXK 2007-0005). Rats were group housed in SPF-grade weather-controlled animal facilities (certificate NO. SYXK 2011-004) (room temperature was maintained at 23 ± 1°C), relative humidity at 60%, and 12 h-light/12 h-dark cycle was applied). Animal experiments were performed according the State Committee of Science and Technology of the People’s Republic of China Order No. 2 on November 14, 1988 (revised in 2011), and the study protocol was approved by the Experimental Animal Ethics Committee of the Zunyi Medical University. All efforts were made to minimize animal use and animal suffering.

### Surgery and Drug Administration

The rats were randomly divided into the following four groups (*n* = 15 for each group): sham group, sham + ICS II group, Aβ group, and Aβ + ICS II group, respectively. The Aβ-induced cognitive impairment in rats was established as previously described (Nie et al., 2010). Briefly, standard aseptic skull drilling procedure was employed in rats after i.p. chloral hydrate (300 mg/kg) anesthesia. Rats then received bilateral hippocampal injection of 5 μL Aβ_25-35_ (2 μg/μL) or 5 μL sterilized NS at the following coordinates: front/rear –3.3 mm, medial/lateral ± 2.0 mm relative to bregma, and dorsal/ventral –3.6 mm below dura. The injection was carried out slowly at a rate of 1 μL/min and left for another 5 min after injection to minimize reflux along the injection track. Sham-operated animals received the same surgery with the exception that sterilized NS was administrated instead of Aβ_25-35_. Rats then received intragastrically administration of ICS II 20 mg/kg when fully awake after surgery, once a day for 15 days or the volume-matched saline according to their group assignments as described above.

### Morris Water Maze

The MWM task was used to assess spatial learning and memory as previously described (Vorhees and Williams, 2005; Gong et al., 2010). Trials were carried out during days 11–15 after the infusion of Aβ_25-35_. The task was conducted in a fixed circular tank (diameter of 120 cm and height of 50 cm), filled with a depth of 30 cm water (23 ± 1°C). Rats were trained to find a clear Perspex platform hidden by arranging its top surface (diameter: 10 cm) 1 cm below the water. The pool was divided into four quadrants with four start positions. The task consisted of two steps. The first step was the place navigation test conducted twice daily for 4 consecutive days, during which the escape latency and the swimming speed were recorded. The animals were allowed 120 s to find the platform and then allowed to remain on the platform for 20 s. If a rat failed to reach the platform within 120 s, it was manually guided to the platform, and remained for 20 s. In this case, its escape latency was marked as 120 s. The second step was run on the 5th day as the spatial probe test without the platform to evaluate the final strength of the memory traces.

### Tissue Preparation

On day 15 after the behavioral tests, animals were anesthetized with 7% chloral hydrate and perfused with 0.1 M PBS. The brains were immediately removed and divided into left and right hemispheres. One hemisphere was fixed in 4% paraformaldehyde for 1 week, and then embedded in paraffin. The other hemisphere separated the hippocampus and stored the hippocampus at -80°C until analysis.

### Hematoxylin and Eosin Staining, Nissl Staining

Embedded brain tissue sections were coronally cut (4 μm) using a Leica slicing machine for HE staining (Li et al., 2015) and Nissl staining (Liu et al., 2015), respectively. The histopathological abnormalities were observed under an optical microscope (KS300, Zeiss-Kontron, Germany).

### TUNEL Assay

TdT-mediated dUTP nick end labeling assay was performed to examine the cellular apoptosis according to the manufacturer’s instructions using the *in situ* cell death detection kit-POD (Roche Diagnostics GmbH Co., Germany). TUNEL staining sections were viewed and counted by a light microscope (KS300, Zeiss-Kontron, Germany). Quantitation was performed by counting the number of positive cells in five randomly chosen fields from each group and the percentage of TUNEL-positive cells was calculated according to this formula: % TUNEL-positive cells = (TUNEL-positive neurons (brown)/total neurons) × 100.

### Immunohistochemical Staining

The brain sections from each animal were microwaved for 5 min, and then incubated with 3% hydrogen peroxide in PBS for 20 min to quench the endogenous peroxidase activity. The samples were then blocked with 10% normal goat serum in PBS for 20 min, and incubated with primary antibody against GFAP (1:500, Abcam, USA) and anti-IBA-1 (1:300, Abcam, USA) overnight at 4°C. After washing with PBS three times, sections were incubated with biotinylated goat anti-rabbit or rabbit anti-goat IgG-horseradish peroxidase (HRP) (1:500, Beyotime, China) for 2 h and then incubated with avidin-biotin peroxidase complex (ImmunoPure ABC kit, Beyotime, China) for 90 min and visualized by a DAB kit (Boster, China).

Immunostained sections were imaged with an Olympus microscope (Olympus, Japan) and assessed using Image Pro Plus 6.0 software (Saur et al., 2014). Results were expressed as the average intensity of the positive immunoreactive cells of GFAP and IBA-1 immunohistochemistry and the average number of immunolabeled cells per mm^2^ in CA1 and dentate gyrus (DG) of the hippocampus. Three digitized images were obtained from each section, and three sections were taken from each animal for analysis. The images were converted to 8-bit gray scales to measure the regional OD and areas of interest (AOIs).

### Quantitative Real-Time PCR

Gene expression of interleukin-1 beta (IL-1β), COX-2, TNF-α and iNOS was detected by real time RT-PCR. Total RNA of hippocampus was extracted using Trizol reagent (Takara Bio Co., China). The primer sequences of the selected genes were designed by the Primer 3 software and listed in **Table 1**. Reverse transcription was undertaken using MuLV reverse transcriptase and Oligo-dT primers (Takara Bio Co., China). Real-time PCR analysis was performed with the SYBR green PCR Master Mix (Takara Bio Co., China). The relative expression levels of the genes were quantified as cycle time (Ct) values normalized with GAPDH of the same sample.

**Table 1:** Primer sequences for real-time RT-PCR analysis.

Gene	GeneBank access	Forward	Reverse
TNF-α	NM_012675.3	TCAGTTCCATGGCCCAGAC	GTTGTCTTTGAGATCCATGCCT
IL-1β	NM_031512.2	GCTGTGGCAGCTACCTATGTCTTG	AGGTCGTCATCATCCCACGAG
COX-2	NM_017232.3	AACACGGACTTGCTCACTTTGTTG	AATGGAGGCCTTTGCCACTG
iNOS	NM_012611.3	ACCTTTATGTTTGTGGCGATG	TCAACCTGCTCCTCACTCAA
GAPDH	NM_017008.4	CAGTGCCAGCCTCGTCTCA	TAACCAGGCGTCCGATACG

### Western Blot Analysis

The protein expression of Aβ_1-40_, COX-2, iNOS, TNF-α, IL-1β, Bax, Bcl-2, and caspase-3 was analyzed using Western blot. Hippocampus were removed from the brain hemispheres and homogenized in radioimmunoassay lysis buffer containing protease inhibitors. The homogenized samples were centrifuged for 15 min at 12,000 *g* and 4°C. Protein concentrations from each sample were determined using the BCA protein assay kit (Biocolor Biotechnology, China). 50 μg protein of each sample was heated at 100°C for 5 min, and then separated by SDS-PAGE and transferred onto a PVDF membrane. The membrane was blocked with 5% non-fat milk in PBS buffer for 2 h, and then incubated with primary antibodies against Aβ_1-40_ (1:1,000, Abcam, USA), IL-1β (1:1,000, Abcam, USA), COX-2 (1:1,000, Abcam, USA), TNF-α (1:1,000, Abcam, USA), iNOS (1:1,000, Abcam, USA), Bax (1:1,000, Abcam, USA), Bcl-2 (1:1,000, Abcam, USA), active + pro caspase-3 (1:1,000, Abcam, USA) and β-actin (1:2,000, Beyotime, China) at 4°C overnight, followed by horseradish peroxidase conjugated secondary antibodies (1:2,000, Beyotime, China) incubation. Immunoblots were visualized with chemiluminescence reagent BeyoECL Plus (Beyotime, China) and quantified with Quantity One software v4.52 (Bio-Rad).

### Statistics

The data were presented as means ± SEM. Water maze test data were analyzed using the repeated measures ANOVA and multivariate tests. Other data were analyzed using one-way ANOVA and followed by Bonferroni’s multiple comparison tests with SPSS 19.0 software. *P* < 0.05 was considered statistically significant.

## Results

### ICS II Alleviated Aβ-Induced Spatial Learning and Memory Impairment

**FIGURE 1:**
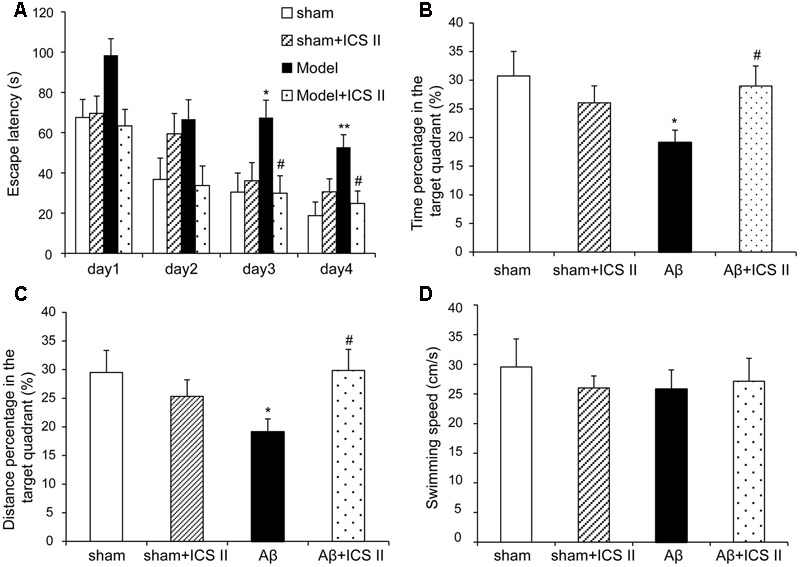
**Icariside II ameliorated Aβ_25-35_-induced learning and memory impairments.** Rats were slowly injected Aβ_25-35_ or vehicle bilaterally into each lateral ventricle and then given 20 mg/kg ICS II for 15 days. Morris water maze was performed for 5 consecutive days from day 11 after surgery. **(A)** The escape latency of the rats to reach the hidden platform from days 1 to 4. **(B)** The time spent in the target quadrant and the frequency crossing the target quadrant. **(C)** The percentage of the time spent in target quadrant on the 5th day. **(D)** The average swimming speed. Data were expressed as mean ± SEM (*n* = 10∼12). ^∗^*P* < 0.05, ^∗∗^*P* < 0.01 vs. sham; ^#^*P* < 0.05 vs. Aβ alone.

To investigate the effect of ICS II on the learning and memory deficits produced by Aβ_25-35_ injection, spatial learning, and memory ability was examined using a 5-day MWM test from day 11 after surgery. As shown in **Figure 1A**, in the navigation test sham and sham + ICS II rats quickly reached the platform, while Aβ_25-35_ treated rats took a longer escape latency to arrive at the location of the hidden platform compared with the sham group starting from day 3 [*F*(3,41) = 4.165, *P* = 0.012], indicating that the memory deficits could be induced by Aβ_25-35_. Escape latency was significantly decreased in ICS II-treated rats compared with that of Aβ_25-35_ group on days 3 and 4 (*P* < 0.05). In the spatial probe test, the time the animals spent in the target quadrant and target quadrant distance in Aβ group were lower than that in sham group (*P* < 0.05, **Figures 1B,C**), indicative of cognitive impairments. However, ICS II significantly increased the time and distance that the Aβ-treated rats spent in target quadrant. Importantly, the swimming speed was no significantly different among these groups, suggesting the lack of motor function deficit (**Figure 1D**). Taken together, these data clearly indicated that ICS II improved Aβ-induced cognitive impairment in rats.

### ICS II Protected against Neuronal Loss and Apoptosis in Aβ_25-35_-Treated Rats

**FIGURE 2:**
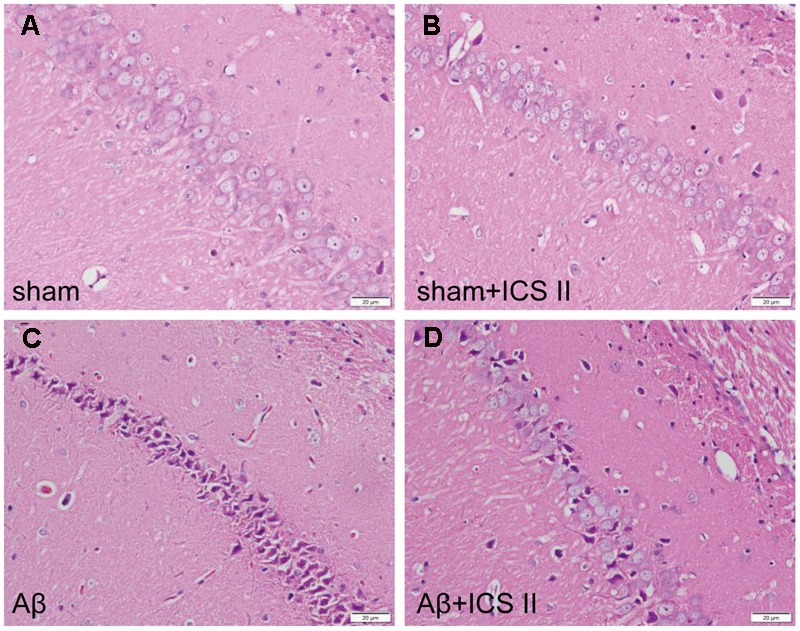
**Icariside II attenuated Aβ_25-35_-induced morphological alterations in the hippocampus. (A–D)** The sections of hippocampus CA1 region were obtained and stained with HE (magnification 400×, scale bar = 20 μm).

Aβ_25-35_-induced neuropathological changes, neuronal loss, and apoptosis were assessed by HE staining, Nissl staining and TUNEL assay, respectively. HE staining (**Figure 2**) showed that the neurons in the sham group had normal morphology and clear boundary, while most hippocampal neurons disappeared, were dark stained or lacking a visible cell boundary in the CA1 areas in the Aβ_25-35_-treated rats compared with those of the sham rats. Moreover, treatment with 20 mg/kg ICS II markedly attenuated the Aβ-induced neuronal damage as evidenced by the fact that few unhealthy cells were observed in ICS II-treated group.

**FIGURE 3:**
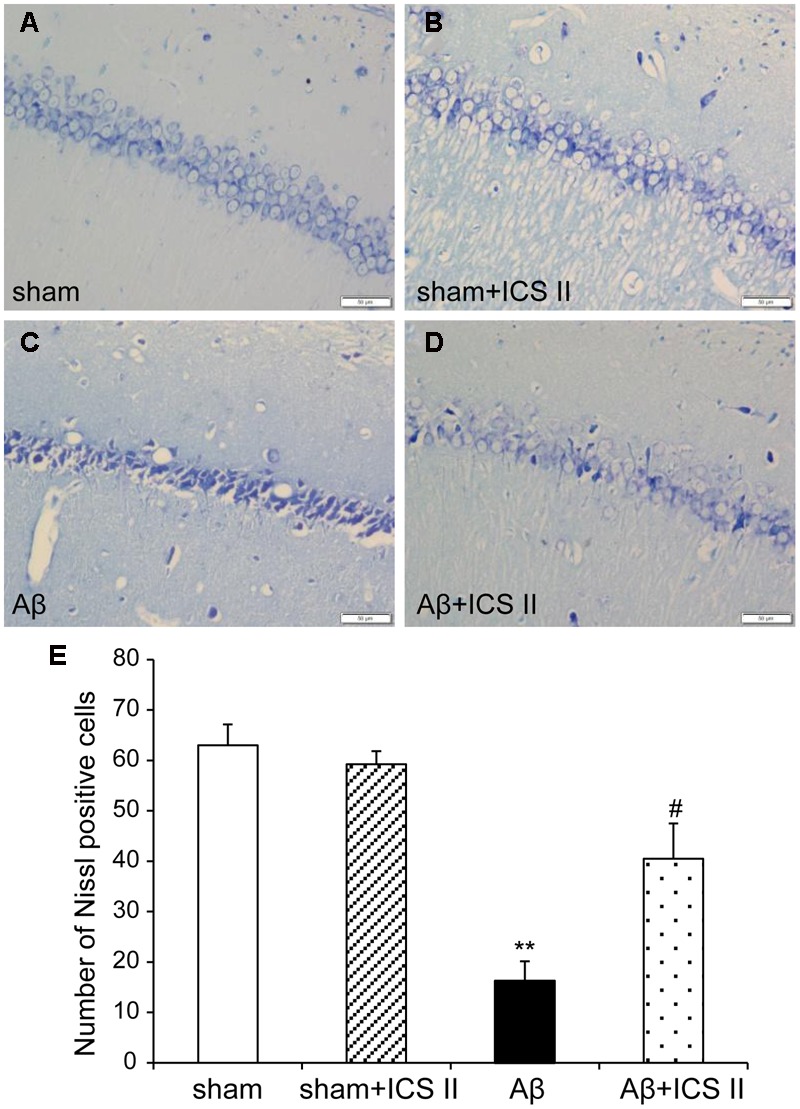
**Icariside II protected against Aβ_25-35_-induced neuronal death in the hippocampus. (A–D)** Nissl staining of hippocampus CA1 region sections (magnification 400×, scale bar 50 μm). **(E)** Quantitative analysis of Nissl bodies in the hippocampus CA1 region. Data were expressed as mean ± SEM, *n* = 4. ^∗∗^*P* < 0.01 vs. sham; ^#^*P* < 0.05 vs. Aβ alone.

Nissl staining (**Figure 3**) showed highly dense pyramidal layer neurons with intact structure in the sham rats. In contrast, the number of neurons was lower and neurons appeared atrophied and pyknotic in the Aβ-treated group [*F*(3,12) = 20.803, *P* < 0.001]. Treatment with ICS II reduced the number of pyknotic and shrunken neurons compared with Aβ alone group (*P* < 0.05).

**FIGURE 4:**
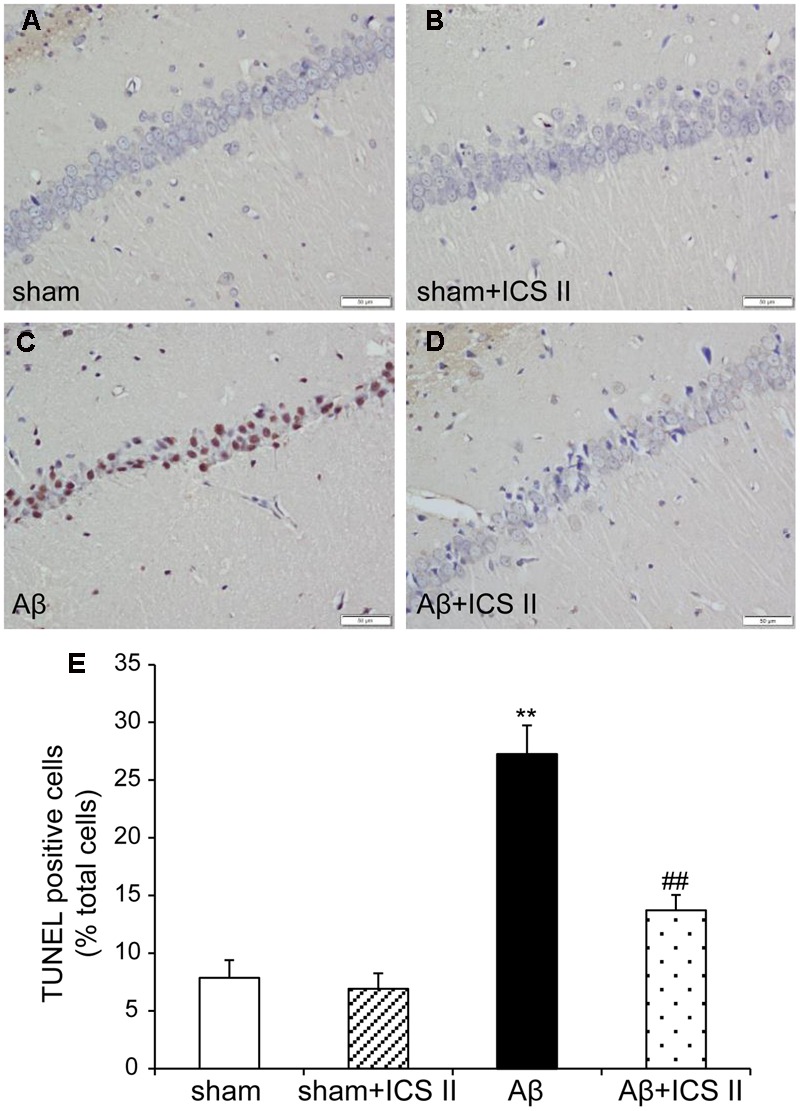
**Icariside II improved Aβ_25-35_-induced neuronal apoptosis in the hippocampus. (A–D)** TUNEL staining of hippocampus CA1 region sections (magnification 400×, scale bar 50 μm) and **(E)** quantitative analysis of apoptotic cells in the hippocampus CA1 region. Data were expressed as mean ± SEM, *n* = 4. ^∗∗^*P* < 0.01 vs. sham; ^##^*P* < 0.01 vs. Aβ alone.

TdT-mediated dUTP nick end labeling assay was performed to further assess the hippocampal neuronal apoptotic damage. Morphologically normal neurons were observed in the hippocampus of the rats in the sham group, suggestive of no TUNEL reaction (**Figure 4**). Rats in Aβ-treated group showed a significant number of TUNEL-stained cell nuclei in the CA1 region of the hippocampus compared with sham group [*F*(3,12) = 28.362, *P* < 0.001], whereas 20 mg/kg ICS II significantly decreased the number of TUNEL-positive neurons (*P* < 0.01). These results demonstrated that treatment with ICS II attenuated neuronal loss and apoptosis in the hippocampal CA1 region induced by Aβ injection.

### ICS II Prevented Aβ Levels in the Hippocampus of Rats

**FIGURE 5:**
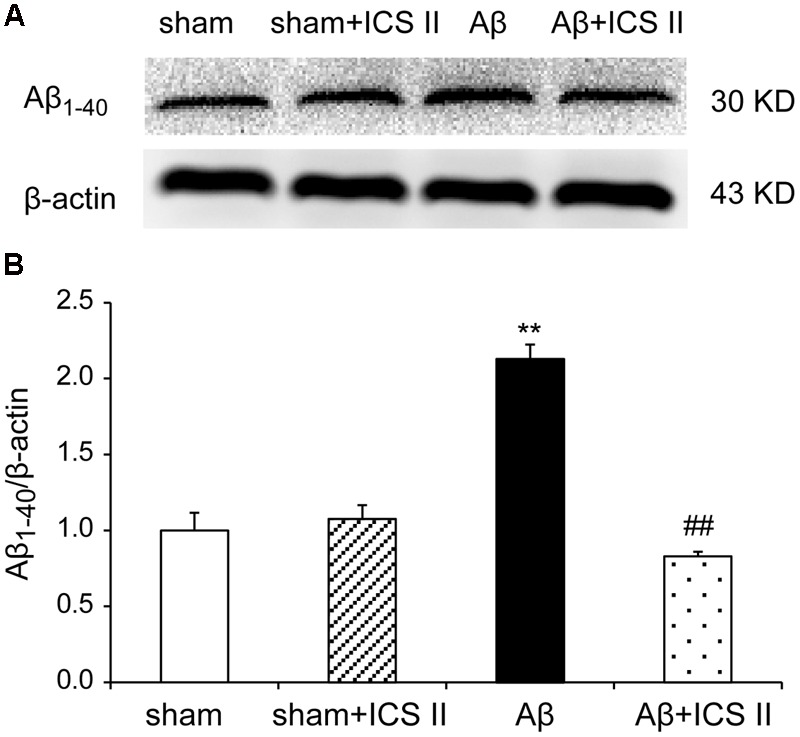
**Icariside II inhibited Aβ_1-40_ levels in the hippocampus of Aβ_25-35_-induced rats.** Aβ_1-40_ levels were examined by Western blot. **(A)** The antibody-reactive band of the Aβ_1-40._ **(B)** Quantitative analysis of Aβ_1-40_ levels. Data were expressed as mean ± SEM (*n* = 3). ^∗∗^*P* < 0.01 vs. sham; ^##^*P* < 0.01 vs. Aβ alone.

Increased production and accumulation of amyloid protein is a key factor in the development of AD pathogenesis (Srivareerat et al., 2011). Contents of Aβ_1-40_ were assessed using Western blot. As indicated in **Figure 5**, the protein levels of Aβ_1-40_ increased in Aβ_25-35_-treated rats compared with those of sham group [*F*(3,8) = 40.084, *P* < 0.001]. However, treatment with ICS II prevented the increase in Aβ_1-40_ levels induced by Aβ_25-35_ (*P* < 0.01).

### ICS II Suppressed Aβ-Induced Astrocytes and Microglial Activation

**FIGURE 6:**
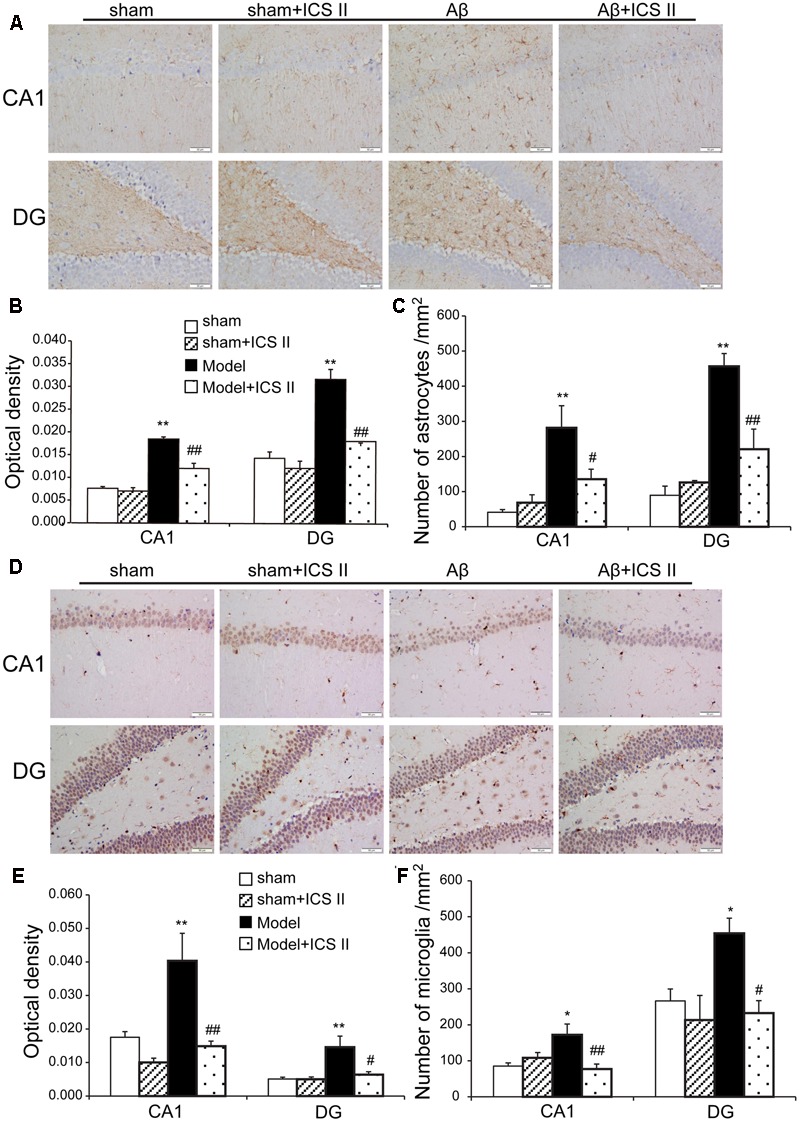
**Icariside II attenuated astrocytes and microglia response after Aβ injection. (A)** GFAP immunoreactivity of hippocampus CA1 and DG region. **(B)** and **(C)** GFAP OD and number of astrocytes. **(D)** IBA-1 immunoreactivity of hippocampus CA1 and DG region. **(E)** and **(F)** IBA-1 OD and number of microglia. Magnification 400×, scale bar = 50 μm. Data were expressed as mean ± SEM, *n* = 4. ^∗^*P* < 0.05, ^∗∗^*P* < 0.01 vs. sham; ^#^*P* < 0.05, ^##^*P* < 0.01 vs. Aβ alone.

Hippocampal injection with Aβ triggers neuroinflammatory responses through the activation of inflammatory cells such as astrocytes and microglia (Diaz et al., 2012). To evaluate the astrocytic and microglia responses, immunohistochemistry staining was used to assess the expression of GFAP (astrocyte activation marker) and IBA-1 (microglia cell activation marker). A significant increase in GFAP-positive staining in the hippocampal CA1 and DG regions from Aβ-induced rats was found as compared to sham group [the average intensity of GFAP-positive in the CA1 and DG regions: *F*(3,12) = 50.214, *P* < 0.001; *F*(3,12) = 31.752, *P* < 0.001; the number of astrocytes in the CA1 and DG regions: *F*(3,12) = 8.702, *P* < 0.001; *F*(3,12) = 20.484, *P* = 0.002, **Figures 6A–C**]. Importantly, both the average intensity of the positive immunoreactive cells and the number of astrocytes were decreased in the CA1 and DG regions of ICS II-treated Aβ rats (*P* < 0.01, 0.01, 0.05, 0.01, respectively). IBA-1 immunostaining showed that injection of Aβ induced the activation of microglia cells as demonstrated by upregulation of IBA-1 expression and the presence of hypertrophic microglia with short thick processes, larger cell body volumes and fewer branches in Aβ_25-35_-treated hippocampus than those in the sham group [the average intensity of IBA-positive in the CA1 and DG regions: *F*(3,12) = 11.618, *P* < 0.001; *F*(3,12) = 8.095, *P* = 0.002; the number of astrocytes in the CA1 and DG regions: *F*(3,12) = 6.365, *P* = 0.006; *F*(3,12) = 4.705, *P* = 0.016, **Figures 6D–F**). However, the number of IBA-immunoreactive microglia was attenuated and the morphology of microglia backed to small cell body possessed long thin branches following ICS II treatment.

### ICS II Attenuated Aβ-Induced Increase of the Expression of Pro-inflammatory Cytokines in the Hippocampus

**FIGURE 7:**
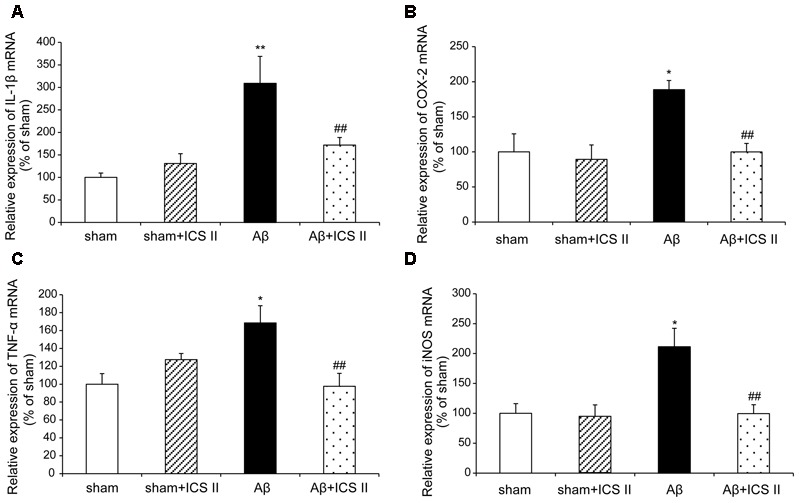
**Icariside II decreased the mRNA expression of TNF-α, IL-1β, COX-2, and iNOS after Aβ injection.** The expression of TNF-α, IL-1β, COX-2, and iNOS mRNA was examined by real time RT-PCR. **(A)** TNF-α mRNA; **(B)** IL-1β mRNA; **(C)** COX-2 mRNA; **(D)** iNOS mRNA. Data were expressed as mean ± SEM (*n* = 6). ^∗^*P* < 0.05, ^∗∗^*P* < 0.01 vs. sham; ^##^*P* < 0.01 vs. Aβ alone.

**FIGURE 8:**
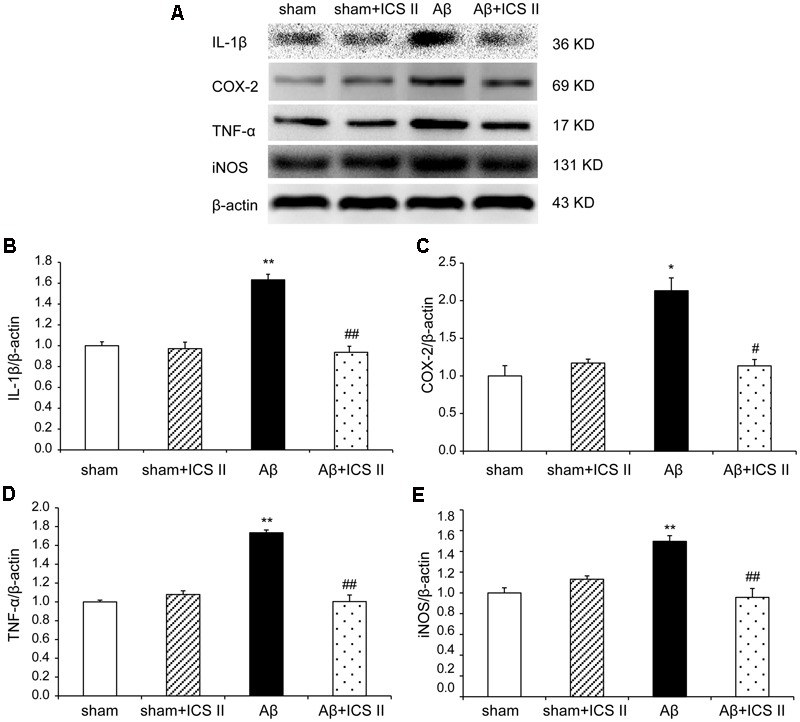
**Icariside II prevented the protein expression of TNF-α, IL-1β, COX-2, and iNOS after Aβ injection.** The expressions of TNF-α, IL-1β, COX-2, and iNOS protein were detected by Western blot analysis. **(A)** The antibody-reactive band of the TNF-α, IL-1β, COX-2, and iNOS, respectively. **(B)** Quantitative analysis of TNF-α protein; **(C)** Quantitative analysis of IL-1β protein; **(D)** Quantitative analysis of COX-2 protein; **(E)** Quantitative analysis of iNOS protein. The relative OD was normalized to β-actin. Data presented as mean ± SEM, *n* = 3. ^∗^*P* < 0.05, ^∗∗^*P* < 0.01 vs. sham; ^#^*P* < 0.05, ^##^*P* < 0.01 vs. Aβ alone.

We then explored whether ICS II affected the generalized pro-inflammatory response from glia. The pro-inflammatory cytokines were assessed by detecting mRNA and protein expression of COX-2, IL-1β, TNF-α and iNOS via real time RT-PCR and Western blot, respectively. Aβ injection dramatically enhanced the expression levels of IL-1β, COX-2, TNF-α and iNOS mRNA, which were 3.09-, 1.89-, 1.69-, and 2.14-fold higher than those of the sham group [IL-1β: *F*(3,20) = 7.303, *P* = 0.002; COX-2: *F*(3,18) = 6.115, *P* = 0.005; TNF-α: *F*(3,20) = 5.473, *P* = 0.007; iNOS: *F*(3,20) = 5.776, *P* = 0.005, **Figures 7A–D**], while treatment with ICS II decreased the mRNA expression of these selected genes (*P* < 0.05, 0.05, 0.01, 0.05, respectively). Consistent with the mRNA expression, injection of Aβ_25-35_ dramatically increased the protein expression levels of IL-1β, COX-2, TNF-α and iNOS [IL-1β: *F*(3,8) = 14.218, *P* = 0.001; COX-2: *F*(3,8) = 9.265, *P* = 0.006; TNF-α: *F*(3,8) = 17.558, *P* = 0.001; iNOS: *F*(3,8) = 10.624, *P* = 0.004, **Figure 8**]. ICS II treatment inhibited the expressions of the above proteins compared with the Aβ group (*P* < 0.01, 0.05, 0.01, 0.01). These data demonstrated that ICS II inhibited the overexpression of inflammatory factors induced by Aβ_25-35_.

### ICS II Repressed the Activation of Apoptotic Signaling in the Hippocampus

**FIGURE 9:**
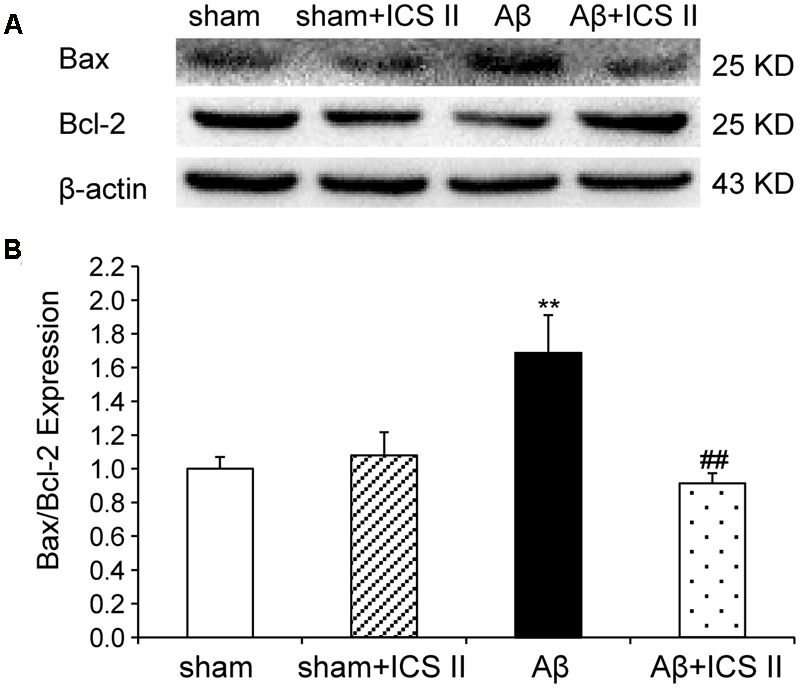
**Icariside II reduced the Bax/Bcl-2 ratio in the hippocampus of Aβ_25-35_-induced rats. (A)** Representative bands of Bax and Bcl-2 of different groups. **(B)** Bax/Bcl-2 ratio. The relative OD was normalized to β-actin. Data were expressed as mean ± SEM (*n* = 3). ^∗∗^*P* < 0.01 vs. sham; ^##^*P* < 0.01 vs Aβ alone.

**FIGURE 10:**
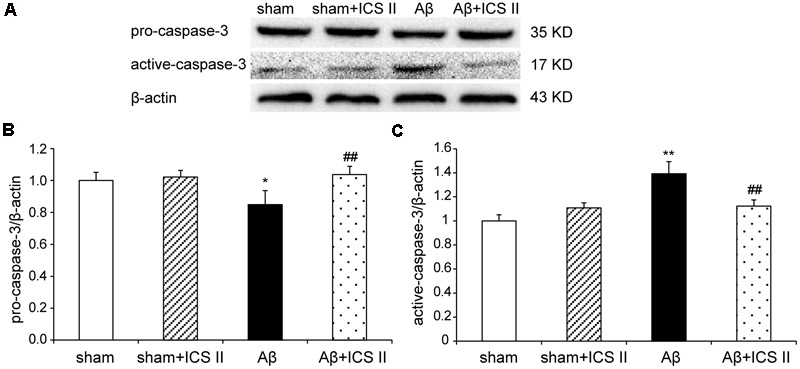
**Icariside II repressed the Aβ-induced activation of caspase-3. (A)** The antibody-reactive band of pro and active-caspase-3. **(B)** Quantitative analysis of pro-caspase-3. **(C)** Quantitative analysis of active-caspase-3. The relative OD was normalized to β-actin. Data were expressed as mean ± SEM (*n* = 3). ^∗^*P* < 0.05, ^∗∗^*P* < 0.01 vs. sham; ^##^*P* < 0.01 vs. Aβ alone.

Because Aβ_25-35_ lowers neuronal cell viability by increasing apoptotic activity (Yao et al., 2007), the expression of Bax, Bcl-2, the pro-caspase-3 and the active caspase-3 were examined to assess whether apoptotic responses were related to the protective effect of ICS II on Aβ-induced neurotoxicity. As shown in **Figure 9**, Aβ injection increased Bax and decreased Bcl-2 and thus enhanced the ratio of Bax to Bcl-2 [*F*(3,8) = 19.308, *P* = 0.001] in the hippocampus as compared to sham group; these effects were significantly reversed by 20 mg/kg ICS II treatment (*P* < 0.01). Meanwhile, Aβ_25-35_ treatment reduced pro-caspase-3 level [*F*(3,8) = 11.180, *P* = 0.003], and ICS II marked reversed the reduction compared with the model group (*P* < 0.05, **Figures 10A,B**). Moreover, the activation of caspase-3 was significantly ameliorated by ICS II treatment [*F*(3,8) = 18.860, *P* = 0.001] (*P* < 0.01, **Figure 10C**). Taken together, the above findings indicated that ICS II suppressed the Aβ_25-35_-induced activation of apoptotic signaling in the hippocampus.

## Discussion

Fibrillar Aβ peptides have been shown to stimulate inflammatory responses (Bona et al., 2010; Abdi et al., 2011), activate the apoptotic pathway (Clementi et al., 2005; Dean et al., 2016), increase neuronal loss (Shie et al., 2003; Thal et al., 2008), and impair spatial learning and memory (Chen et al., 2000; Wei-Na et al., 2013). Aβ production and its clearance are dynamically balanced in the normal brain. Aβ generates from the amyloid precursor protein (APP) and clearance through enzymatic degradation pathways, microglial phagocytosis Aβ and cell autophagy, etc. (Yoon and Ahn Jo, 2012; Kruppa et al., 2013). When the balance is broken, there will be an increase in Aβ levels and trigger aggregation and deposition of Aβ in the brain, eventually lead to memory impairment (Christoph et al., 2003; Jun-Xia et al., 2013). The Aβ peptides of 40 and 42 amino acids are prominent species in the senile plaques of patients, where Aβ_1-40_ is more abundant, Aβ_1-42_ shows stronger neurotoxicity. However, Aβ_25-35_ is also a toxic fragment of the full-length Aβ peptides and it has been demonstrated that Aβ_1-42_ and the Aβ_25-35_ peptides induce neural injury in a similar pattern and Aβ_25-35_ is a convenient tool for the investigation of neurotoxic mechanisms involved in AD (Miao et al., 2008; Frozza et al., 2009). Hence in the present study, we also used Aβ_25-35_ to mimic the pathology and symptoms of AD, and revealed that bilateral Aβ_25-35_ injection into rat’s hippocampi could induce learning and memory deficits. CA1 subfield of the hippocampus is the most damaged area in the brain of rats received hippocampal injection of Aβ_25-35_ (Stepanichev et al., 2004). The present study consistently demonstrated that neuronal degeneration and death were evident in the CA1 region of the hippocampus of Aβ_25-35_-induced rats (Supplementary Figures 1–3).

Icariside II and icariin are the two major active ingredients of *Herba Epimedii* with close structural relationship, icariin has been reported as the most valuable compound of *Epimedii* to be developed to a promising neuroprotective drug treating AD, ischemia and vascular dementia (VD) (Urano and Tohda, 2010; Jin et al., 2014; Li et al., 2014). ICS II is one of the metabolites of icariin and is widely reported as a broad-spectrum anti-cancer agent in recent years (Cai et al., 2011; Arief et al., 2015). Our previous studies showed that ICS II has neuroprotective effects during cerebral ischemia-reperfusion via inflammatory inhibition (Deng et al., 2016). Interestingly, the present study demonstrated administration of ICS II at the dose of 20 mg/kg markedly ameliorated the cognitive deficits and neuronal damage in the Aβ_25-35_ rats. That is to say, in addition to anti-cancer effects, ICS II also showed beneficial neuroprotective effects. It has been proposed that icariin could decrease the generation of endogenous Aβ in hippocampus of rats induced by exogenous injection of Aβ_25-35_. In the present study, the level of Aβ_1-40_ in the hippocampus was significantly higher in the Aβ_25-35_-induced rats than vehicle-treated rats. However, the contents of Aβ_1-40_ were reduced after treatment of ICS II in Aβ_25-35_ rats, indicating ICS II might exert the neuroprotective effects in a similar manner with icariin.

Neuroinflammation contributes to the pathogenesis of numerous neurodegenerative diseases, including AD (Eikelenboom et al., 2010; Reale et al., 2010). Injection of Aβ triggers activation of microglia and astrocytes, which can release inflammatory cytokines and mediators (Matsuoka et al., 2001; Sastre et al., 2006). In the brains of AD, elevated levels of IL-1β and TNF-α suppress phagocytosis of Aβ thereby impeding efficient plaque removal by resident microglia, which in turn augments the inflammatory response and formation of Aβ (Tuppo and Arias, 2005; Morales et al., 2010). Several studies have shown that COX-2 was overexpressed in AD rat brain induced by Aβ_25-35_ and cerebrospinal fluid of AD patients (Minghetti, 2004; Cheng et al., 2006). Similarly, Aβ injection increases the expression of iNOS (Huang et al., 2011), suggesting that iNOS is a mediator of the inflammatory cascade of AD. The present study demonstrated that Aβ_25-35_ injection into the hippocampus activated microglia and astrocytes and then enhanced the expression of TNF-α, IL-1β, COX-2, and iNOS, accompanied with an elevated endogenous Aβ level. Part of the reason may lie in that exogenous injection of Aβ_25-35_ initiates chronic inflammation response. Chronic inflammation inhibits the activation of phagocytic machinery and affects the ability of microglia to mount a phagocytic response, thereby inhibiting clearance of Aβ from microglia (Kruppa et al., 2013), and then break the balance of Aβ production and degradation, which in turn lead to a generation of endogenous Aβ. However, the Aβ-induced neuroinflammatory responses were largely attenuated by ICS II treatment, which is consistent with our previous findings that administration with ICS II suppression the level of Aβ and inhibition of inflammation in streptozotocin (STZ)-induced rats (Yin et al., 2016). Therefore it is postulated that ICS II might exhibit a neuroprotective effect by reducing Aβ concentration and suppressing the neuroinflammatory response in the hippocampus, thus preventing cell death and improving spatial learning.

Biochemical and morphological evidences of *in vitro* and *in vivo* studies have confirmed that apoptotic pathway is involved in the pathogenesis of AD (LeBlanc, 2005). Given the fact that hippocampus neuronal apoptosis is one of the major causes of memory loss accompanied by neurodegeneration (Mattson, 2000), cell apoptosis was further investigated. TUNEL assay displayed a significant number of TUNEL positive cells in the hippocampus of Aβ_25-35_-treated animals. In agreement with the effect of neuroprotection, ICS II treatment significantly decreased the apoptotic cells in Aβ_25-35_ treated rats. Various molecules have been found to participate in the mechanism of apoptosis, and the Bcl-2 family members are the most studied molecules in the regulation of apoptosis (Kitamura et al., 1998; Czabotar et al., 2014). Bcl-2 is an anti-apoptotic member of Bcl-2 family and Bax is a pro-apoptotic member working in a manner opposite to that of Bcl-2. The ratio of Bax/Bcl-2 has been considered a critical factor to determine cell survival and death. Microinfusions of Aβ_25-35_ down-regulated Bcl-2 and up-regulated Bax, leading to the elevation of the Bax/Bcl-2 ratio have been widely reported. Our results are in agreement with previous studies indicating that Bcl-2 family proteins regulate apoptosis following Aβ insult and further demonstrated that ICS II treatment attenuated the hippocampal neuronal apoptosis by decreasing the ratio of Bax/Bcl-2. Moreover, intracellular apoptotic cascade including caspases family can be triggered which leads to the increases in apoptotic responses under Aβ toxicity (Louneva et al., 2008). The present results provided additional data showing that intracerebral injection of Aβ_25-35_ decreased the level of pro-caspase-3 and increased the level of active-caspase-3 in the hippocampus. However, ICS II markedly inhibited the activation of caspase-3 in Aβ-induced rats. Taken together, these data suggest that ICS II might serve as a potent anti-apoptotic agent, which can inhibit the apoptotic responses induced by Aβ_25-35_ and produce anti-inflammatory effects.

It is well known that no matter in AD brain or tumor microenvironment, NF-κB is a crucial transcriptional factor involved in apoptosis and inflammatory responses. It is reported that ICS II might prohibit cancer invasion and metastasis in inflammatory microenvironment through inactivating NF-κB pathway (Jie et al., 2016). Consistent with its effects on cancer, our previous study showed that ICS II protected against cerebral ischemic/reperfusion injury via inhibiting IκBα degradation and the subsequent phosphorylation of NF-κB, by which elevated levels of inflammatory cytokines are repressed (Deng et al., 2016). Thus, we speculate that the suppression of NF-kB signaling might be involved in the protective effects of ICS II on cognitive impairment and associated neuroinflammation and apoptosis. In addition, transgenic model of AD will be further utilized to clarify the underlying mechanisms of ICS II action on AD in more depth.

## Conclusion

In summary, the present study demonstrates that ICS II protects against learning and memory impairments and neuronal death in the hippocampus induced by Aβ_25-35_ in rats. The neuroprotective mechanisms may be attributable to the blockade of inflammation and apoptosis. These results suggest that ICS II might be a promising therapeutic agent for the treatment of AD.

## Author Contributions

QG and YD developed the original idea, wrote the manuscript, and acted as guarantor. YD, KW and LL performed animal studies and behavioral experiments. YD, LZ, LH, and JZ conducted the morphology experiments. YD carried out the RT-PCR. LL performed the Western Blot assay. All authors read and approved the final manuscript.

## Conflict of Interest Statement

The authors declare that the research was conducted in the absence of any commercial or financial relationships that could be construed as a potential conflict of interest.

## Abbreviations

AD: Alzheimer’s disease
ANOVA: analysis of variance
Aβ: beta-amyloid
BCA: bicinchoninic acid
COX-2: cyclooxygenase-2
GFAP: glial fibrillary acidic protein
HE: hematoxylin-eosin
IBA-1: ionize calcium-binding adapter molecule 1
ICS II: Icariside II
IL-1β: interleukin-1β
iNOS: inducible nitric oxide synthase
MWM: Morris water maze
NS: normal saline
OD: optical density
PBS: phosphate buffered saline
TNF-α: tumor necrosis factor
TUNEL: TdT-mediated dUTP nick end labeling
