# Supplementary material for: Icariside II, a Broad-Spectrum Anti-cancer Agent, Reverses Beta-Amyloid-Induced Cognitive Impairment through Reducing Inflammation and Apoptosis in Rats
Source: Front Pharmacol. 2017 Feb 2;8:39. doi: 10.3389/fphar.2017.00039 (PMC5288340; doi:10.3389/fphar.2017.00039)
Supplement: Supplementary file 1 [file Data_Sheet_1.DOCX]

Supplementary Material

Icariside II, a broad-spectrum anti-cancer agent, reverses beta-amyloid-induced cognitive impairment through reducing inflammation and apoptosis in rats

Yuanyuan Deng, Long Long, Keke Wang, Jiayin Zhou, Lingrong Zeng, Lianzi He, Qihai Gong*

*** Correspondence:** Qihai Gong: gqh@zmc.edu.cn

## Supplementary Figures


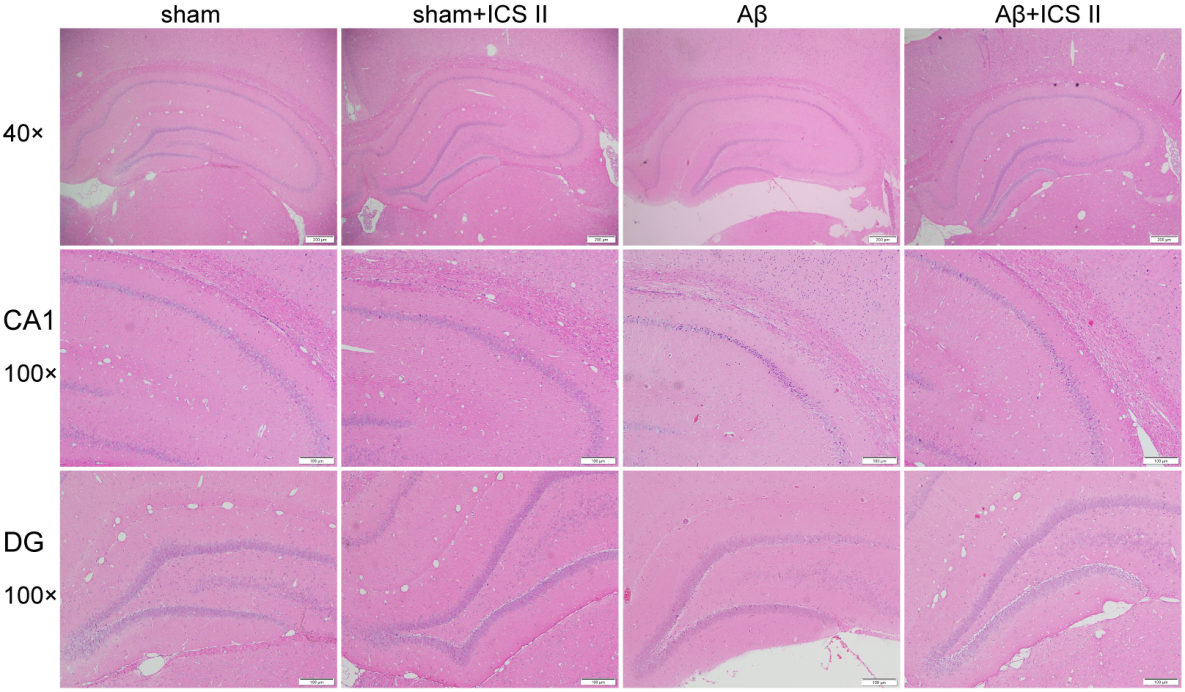


**Supplementary Figure 1.** ICS II attenuated Aβ_25-35_-induced morphological alterations in the hippocampus. The sections of hippocampus CA1 and DG regions were obtained and stained with HE (magnification 40× and 100×, scale bar = 200 μm and 100 μm).


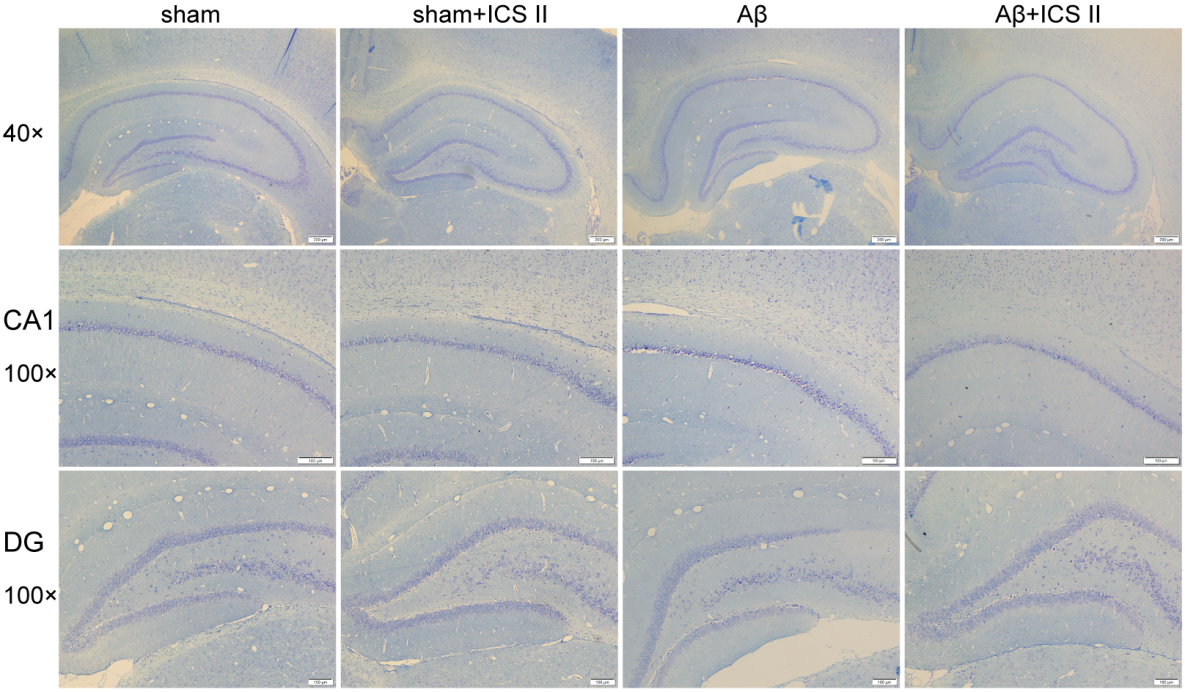


**Supplementary Figure 2.** ICS II protected against Aβ_25-35_-induced neuronal death in the CA1 subfield of the hippocampus. Nissl staining of hippocampus CA1 and DG regions (magnification 40× and 100×, scale bar = 200 μm and 100 μm).


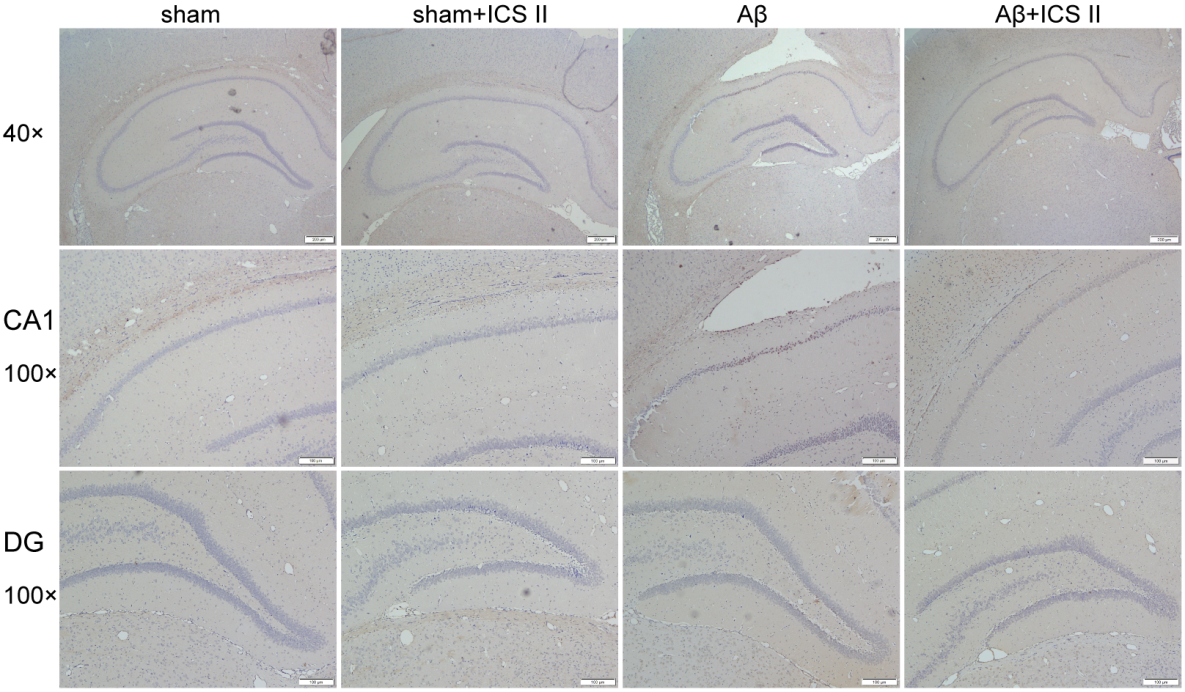


**Supplementary Figure 3.** ICS II improved Aβ_25-35_-induced neuronal apoptosis in the CA1 region of the hippocampus. TUNEL staining of hippocampus CA1 and DG regions (magnification 40× and 100×, scale bar = 200 μm and 100 μm).
